# The relationship between frequent premature ventricular complexes and epicardial adipose tissue volume

**DOI:** 10.3389/fendo.2023.1219890

**Published:** 2023-09-26

**Authors:** Zhe Wang, Siqi Jiao, Jiawei Chen, Hehe Guo, Lichen Ren, Liping Sun, Yihong Sun, Yingwei Chen

**Affiliations:** ^1^ Department of Cardiology, The First Affiliated Hospital of Zhengzhou University, Zhengzhou, China; ^2^ Department of Cardiology, Peking University China-Japan Friendship School of Clinical Medicine, Beijing, China; ^3^ Department of Radiology, The First Affiliated Hospital of Zhengzhou University, Zhengzhou, China

**Keywords:** premature ventricular complexes, epicardial adipose tissue, computed tomography, burden levels, propensity score matching

## Abstract

**Background:**

Epicardial adipose tissue (EAT) is related to atrial fibrillation. The association between EAT volume and premature ventricular complexes (PVCs) remains unclear. Our study aimed to investigate the effect of EAT volume on the risk of frequent PVCs and burden levels of PVCs.

**Methods:**

This observational study retrospectively recruited consecutive patients who had consultation between 2019 and 2021 at the First Affiliated Hospital of Zhengzhou University. Frequent PVC patients (*n* = 402) and control patients (*n* = 402) undergoing non-contrast computed tomography (CT) were enrolled. We selected evaluation criteria for the conduct of a 1:1 propensity score matching (PSM) analysis. Multivariable logistic analysis was used to investigate factors related to frequent PVCs. Furthermore, the determinants of EAT volume and the burden levels of PVCs were evaluated.

**Results:**

Patients with PVCs had a significantly larger EAT volume than control patients. EAT volume was significantly larger in male PVC patients with BMI ≥24 kg/m^2^, diabetes mellitus, and E/A ratio <1. EAT volume was independently associated with PVCs. Moreover, the larger EAT volume was an independent predictor for the high burden level of PVCs. We revealed that the risk of high PVC burden level was increased with the rising of EAT volume by restricted cubic splines.

**Conclusions:**

EAT volume was larger in frequent PVC patients than in control patients, regardless of other confounding factors. A large EAT volume was independently associated with high burden levels of PVCs. EAT volume may be a new mechanism to explain the pathogenesis of PVCs.

## Introduction

Premature ventricular complexes (PVCs) are common diseases in clinical practice. The burden levels of PVCs are one of the most important indicators of PVC risk (1). Frequent PVCs are usually accompanied by symptoms and are thought to cause arrhythmia-induced cardiomyopathy (2). The risk factors associated with frequent PVC have not been completely revealed (3).

Epicardial adipose tissue (EAT) is an adipose tissue that accumulates between the epicardium and myocardium. EAT is 20% of the heart mass under physiologic conditions (4). EAT can be considered an endocrine organ with local effects on the heart through vasocrine or paracrine secretion of pro-inflammatory and profibrotic cytokines, which promote the development of arrhythmias (5). One study found that large pericardial fat is strongly associated with ventricular arrhythmias in 50 patients with heart failure (6). Another study revealed that EAT thickness is increased, by transthoracic echocardiography measurement, in 50 patients with frequent PVCs, compared with control subjects (7). However, the role of EAT in PVCs remains unclear. The purpose of this study was to investigate the relationship between EAT volume and frequent PVCs.

## Methods

### Study population

This observational study retrospectively recruited consecutive patients who had consultation between January 2019 and December 2021 at the Department of Cardiology, First Affiliated Hospital of Zhengzhou University. Data were extracted from the hospital’s files and were anonymized. The study population consisted of patients diagnosed with PVCs and presented to our hospital for routine follow-up. The inclusion criteria were as follows: 1) patients who underwent non-contrast CT, 2) patients symptomatic with frequent PVCs (>10,000/24 h), 3) patients >18 years of age, and 4) absence of severe valvular heart disease and hypertrophic heart disease. The exclusion criteria were follows: 1) patients with a history of prior radiofrequency ablation, 2) patients with poor/insufficient CT images, 3) patients with atrial fibrillation (AF), and 4) patients with coronary heart disease, including angina pectoris, myocardial infarction, percutaneous coronary intervention, and coronary artery bypass surgery. The control group (*n* = 402) matched with the PVC patients during the same time period, which had no detected PVCs using the 24-h Holter monitoring. Patients with a diagnosis of PVCs were identified using the International Classification of Disease (ICD) code in our hospital’s electronic health record systems. Frequent PVC was defined as a total PVC count of >10,000 beats during a 24-h Holter monitoring. Non-contrast CT was performed for patients who wanted examination for various reasons including smoking histories, shortness of breath, or screening for lung disease in our institution. The study complied with the Declaration of Helsinki. The study protocol was authorized by the local institution’s ethics committee and waived the need for written informed consent (2022-KY-0288).

### Clinical and laboratory data

The following data were collected from all patients: demographic parameters, comorbidities, echocardiography parameters [left ventricular ejection fraction (LVEF), left atrial diameter (LAD), E/A ratios], CT parameter (EAT volume), and burden levels of PVCs on admission. PVC burden = total ventricular premature beats/total beats × 100%. Body mass index (BMI) ≥24 kg/m^2^ was defined as overweight or obese. Smokers and drinkers were either former or active smokers and drinkers.

### CT acquisition

All individual examinations use a dual-source CT system (Somatom Force, Siemens Healthineers, Forchheim, Germany). Non-contrast CT was performed with 120 kV, and the tube current depended on body habitus and heart rate. The images were reconstructed with a slice thickness of 0.5 mm, a reconstruction increment of 0.5 mm with a medium soft-tissue convolution kernel (B26F), and a reconstructed matrix size of 512 × 512.

### EAT volume quantification

**Figure 1:**
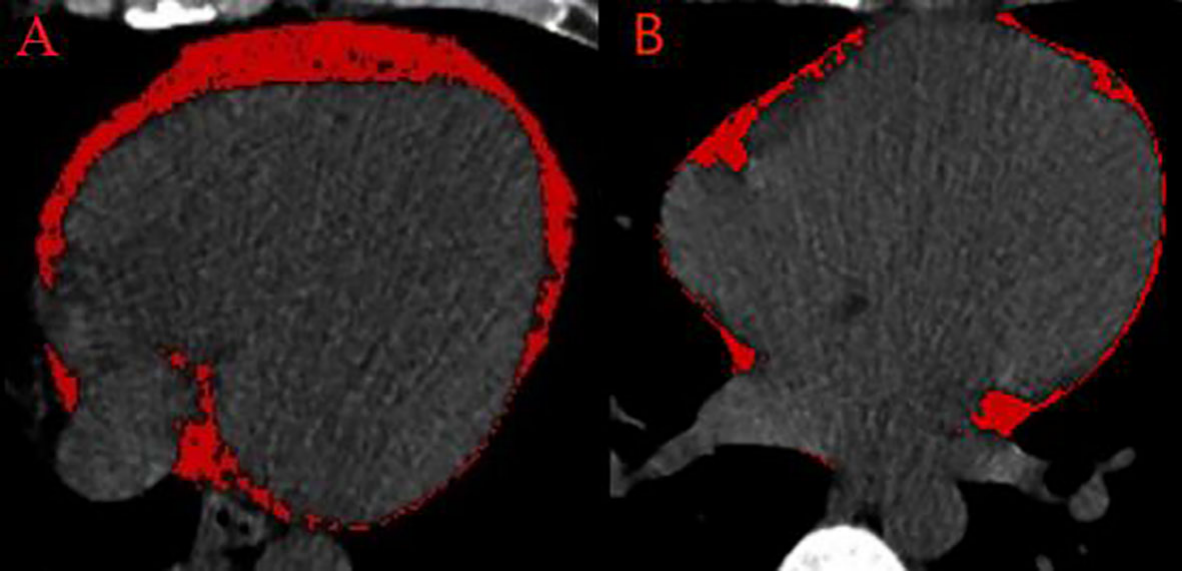
Semiautomated EAT volume analysis on non-contrast CT. **(A)** A patient with frequent PVCs. **(B)** A control patient. CT, computed tomography; EAT, epicardial adipose tissue; PVCs, premature ventricular complexes.

EAT volume was quantified using a dedicated semiautomatic software (syngo.via Frontier Cardiac Risk Assessment, version 1.2.3, Siemens Healthineers, Germany), as shown in **Figure 1**. Previous studies used the sophisticated software to quantify EAT volume (8–10). EAT is defined as non-contrast CT density ranging from −195 Hounsfield units (HU) to −45 HU (8, 10, 11). The software automatically delineates and identifies EAT and manually adjusts the contour of EAT volume if necessary. EAT volume measurement was performed by two independent radiologists who were unaware of the patient’s clinical data.

### Statistical analysis

The values of the continuous variables were described as the mean ± standard deviation or median (Q1, Q3 quartiles) and compared among the groups using Student’s *t*-test or Mann–Whitney *U* test, depending on whether the data were normally distributed. Categorical variables are presented as numbers (percentage) and compared between two groups by the Pearson chi-squared test or Fisher exact test. The propensity score matching (PSM) 1:1 was applied in the observational case–control study to reduce bias in selecting the case controls. Matching of the two groups was performed for basic characteristics (age, sex), and variables subjected to univariate analysis showed *p <*0.10 (BMI ≥ 24 kg/m^2^, hypertension, diabetes mellitus, fasting blood glucose, total cholesterol, E/A ratio <1, LVEF, and LAD). We calculated the standardized mean difference to assess the balance of baseline characteristics after PSM. Receiver operating characteristic (ROC) curve analysis was performed to analyze discriminative power, specificity, and sensitivity to predict the frequent PVC risk based on the EAT volume.

The associations between EAT volume and continuous variables were determined using Pearson’s correlation analysis. The cutoff values of the PVC burden levels were determined by the median index. Variables subjected to univariable analysis showed *p*-value <0.10, and important clinical risk factors were included in the multivariable analysis. Multivariable logistic analysis was performed to investigate factors associated with PVC risk and burden levels after adjusting other confounders. We also used restricted cubic splines (RCS) with four knots at the 5th, 25th, 75th, and 95th centiles to flexibly model the relationship between EAT volume and PVC burden levels. Furthermore, we compared EAT volume by sextile of the PVC burden levels, with the first sextile serving as the reference. Statistical analysis was performed using R language version 4.0.3 (R Foundation for Statistical Computing, Vienna, Austria). A two-sided *p*-value of <0.05 was considered statistically significant.

## Results

### Baseline clinical features

Four hundred ninety-nine frequent PVC patients were screened for eligibility. Four hundred and two PVC patients were included in the final analysis, as shown in **Supplementary Figure 1**. The gap time between the non-contrast CT and the 24-h Holter monitoring scan performed was 2 (1–3) days in frequent PVC patients. The patients with frequent PVCs had significantly higher proportions of hypertension, diabetes mellitus, E/A ratio <1, and BMI ≥24 kg/m^2^, compared with control patients. In the unadjusted cohort, PVC patients also had significantly larger age, BMI, fasting blood glucose, total cholesterol, LVEF, and LAD, compared with the control patients.

PSM was used to reduce bias in the selection of potential clinical confounders. Following the 1:1 matching protocol, differences in clinical characteristics between the two groups were eliminated. Compared with the control patients, patients’ age, hypertension, diabetes mellitus, E/A ratio <1, BMI ≥24 kg/m^2^, BMI, fasting blood glucose, total cholesterol, LVEF, and LAD showed no significant differences between the two groups in the PSM cohort, as shown in **Table 1**. Based on the propensity score, PSM achieved an optimal balance between the two groups. The standardized mean difference values were all under 0.1, which indicated that confounding factor bias was attenuated (**Supplementary Figure 2**).

**Table 1:** Baseline demographics and clinical characteristics of the study patients.

Variables	Unadjusted cohort			PSM cohort		
	PVCs (*n* = 402)	Control (*n* = 402)	*p-*value	PVCs (*n* = 294)	Control (*n* = 294)	*p-*value
Age, years	47.9 ± 12.2	45.7 ± 12.6	0.012	46.4 ± 12.4	46.8 ± 12.4	0.687
Female	209 (51.7)	194 (48.0)	0.290	155 (52.7)	149 (50.7)	0.620
BMI ≥ 24 kg/m^2^	242 (60.2)	207 (51.5)	0.013	161 (54.8)	165 (56.1)	0.740
BMI, kg/m^2^	24.9 ± 3.4	24.3 ± 4.0	0.049	24.4 ± 3.2	24.8 ± 4.0	0.260
Smoker	67 (16.7)	57 (14.3)	0.344	49 (16.7)	41 (14.1)	0.388
Drinker	59 (14.7)	60 (14.9)	0.921	45 (15.3)	43 (14.6)	0.817
Hypertension	66 (16.4)	37 (9.2)	0.002	32 (10.9)	35 (11.9)	0.697
DM	33 (8.2)	19 (4.7)	0.045	16 (5.4)	15 (5.1)	0.854
WBC, 10^9^/L	6.3 ± 1.7	6.2 ± 1.9	0.375	6.3 ± 1.7	6.2 ± 1.8	0.390
FPG, mmol/L	5.0 ± 1.3	4.8 ± 1.0	0.023	4.8 ± 1.0	4.9 ± 1.1	0.564
TC, mmol/L	4.3 ± 0.7	4.0 ± 0.8	<0.001	4.2 ± 0.7	4.2 ± 0.8	0.366
TG, mmol/L	1.2 (0.9–18)	1.2 (0.8–1.7)	0.222	1.2 (0.8–1.7)	1.3 (0.8–1.7)	0.443
UA, μmol/L	297.4 ± 88.0	289.8 ± 94.7	0.241	289.8 ± 80.2	293.9 ± 97.0	0.577
HS-CRP (>2 mg/L)	48 (11.9)	40 (10.0)	0.187	35 (13.8)	30 (11.6)	0.465
LVEF	62.4 ± 3.5	63.0 ± 1.5	0.001	62.9 ± 3.2	63.0 ± 1.5	0.634
LAD, mm	33.6 ± 5.9	30.7 ± 2.6	<0.001	31.4 ± 3.7	31.2 ± 2.6	0.415
E/A ratio <1	199 (49.5)	162 (40.3)	0.009	131 (44.6)	134 (45.6)	0.804
EAT volume, ml	135.6 ± 64.3	103.4 ± 56.0	<0.001	126.6 ± 60.5	109.7 ± 58.2	0.001

BMI, body mass index; DM, diabetes mellitus; EAT, epicardial adipose tissue; FPG, fasting blood glucose; HS-CRP, high-sensitivity C-reactive protein; LAD, left atrial diameter; LVEF, left ventricular ejection fraction; TC, total cholesterol; TG, triglycerides; PSM, propensity score matching; PVCs, premature ventricular complexes; UA, uric acid; WBC, white blood cell.

### EAT volume characteristics among PVC patients

**Figure 2:**
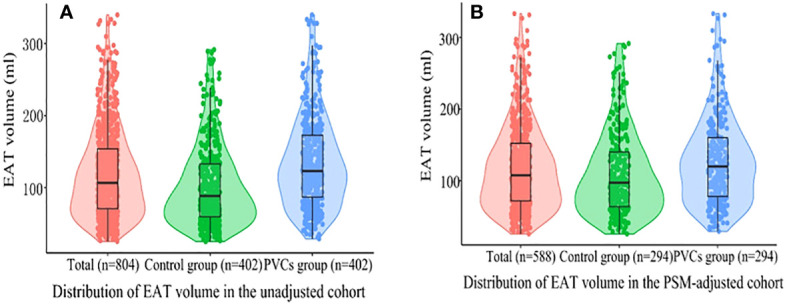
Violin plots of the distribution of EAT volume. **(A)** All patients in the unadjusted cohort; **(B)** all patients in the PSM cohort. Different color dots represent measured EAT volume; the top of the box, the 75th percentile; the horizontal line, the 50th percentile (median); the bottom of the box, the 25th percentile; the line represents the distribution area of the 95% confidence interval of the data. EAT, epicardial adipose tissue; PSM, propensity score matching; PVCs, premature ventricular complexes.

**Figure 3:**
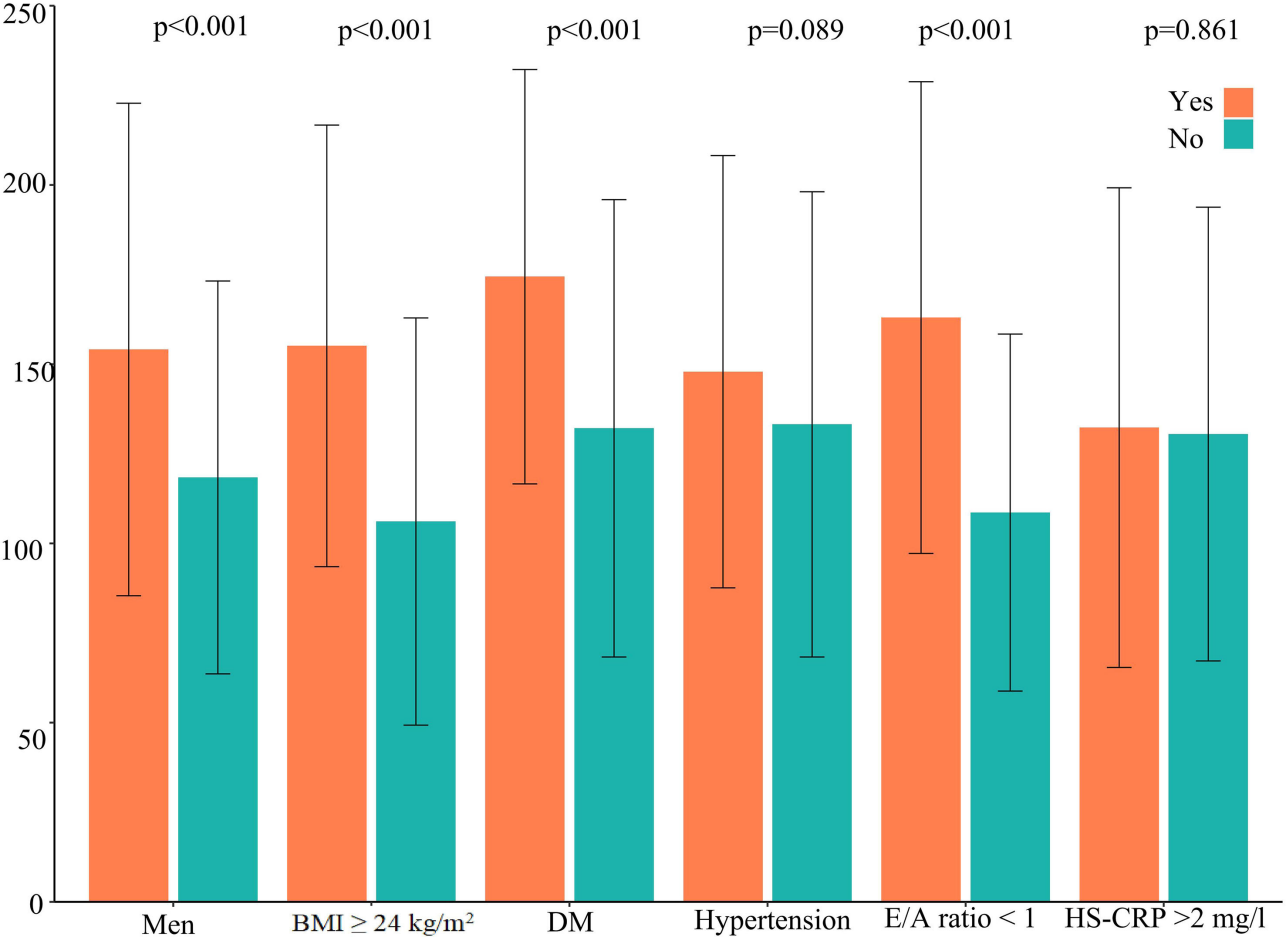
EAT volume in frequent PVC patients according to cardiovascular risk factors. DM, diabetes mellitus; HS-CRP, high-sensitivity C-reactive protein; EAT, epicardial adipose tissue; PVCs, premature ventricular complexes.

The distribution of EAT volume is shown in **Figure 2**. PVC patients had significantly larger EAT volume compared with control patients in the unadjusted cohort and PSM cohort. The cutoff points of EAT volume for the prediction of frequent PVCs were calculated as 102.81 ml, and the AUC was 0.660 (95% CI: 0.622–0.697). The specificity and sensitivity were 60.2% and 66.9%, respectively, as depicted in **Supplementary Figure 3**. EAT volume was significantly correlated with age (*r* = 0.396, *p* = 0.001), BMI (*r* = 0.388, *p* < 0.001), and LAD (*r* = 0.322, *p* < 0.001) in the 402 PVC patients (**Supplementary Table 1**). EAT volume was significantly larger in male PVC patients with BMI ≥24 kg/m^2^, diabetes mellitus, and E/A ratio <1, as described in **Figure 3**. Notably, the multivariable logistic analysis found that EAT volume (per 10 ml increase) was an independently associated factor for PVCs after adjusting different confounders, as indicated in **Table 2**.

**Table 2:** Association between EAT volume and frequent PVCs.

Variables	Unadjusted cohort		PSM cohort	
	OR (95% CI)	*p-*value	OR (95% CI)	*p-*value
Crude model (no adjustment)	1.10 (1.07–1.12)	<0.001	1.05 (1.02–1.08)	0.001
Adjusting for age and sex	1.12 (1.08–1.15)	<0.001	1.07 (1.04–1.11)	<0.001
Adjusting for age, sex, and BMI	1.13 (1.09–1.17)	<0.001	1.10 (1.06–1.14)	<0.001
Adjusting for variables^a^	1.13 (1.09–1.16)	<0.001	1.09 (1.05–1.14)	<0.001
Adjusting for variables^b^	1.08 (1.04–1.11)	<0.001	1.09 (1.05–1.13)	<0.001
Adjusting for variables^c^	1.10 (1.06–1.14)	<0.001	1.10 (1.06–1.14)	<0.001

Variables^a^ included age, sex, BMI, smoker, and drinker; variables^b^ subjected to univariate analysis of the unadjusted cohort showed p-value <0.10, which included age, hypertension, DM, BMI, TC, FPG, LAD, LVEF, and E/A ratio <1; variables^c^ that included variables^a^ and variables^b^, which included age, sex, BMI, smoker, drinker, hypertension, DM, TC, FPG, LAD, LVEF, and E/A ratio <1.

BMI, body mass index; DM, diabetes mellitus; EAT, epicardial adipose tissue; FPG, fasting blood glucose; LAD, left atrial diameter; LVEF, left ventricular ejection fraction; TC, total cholesterol; PSM, propensity score matching; PVCs, premature ventricular complexes.

### EAT volume and burden levels of PVCs

**Figure 4:**
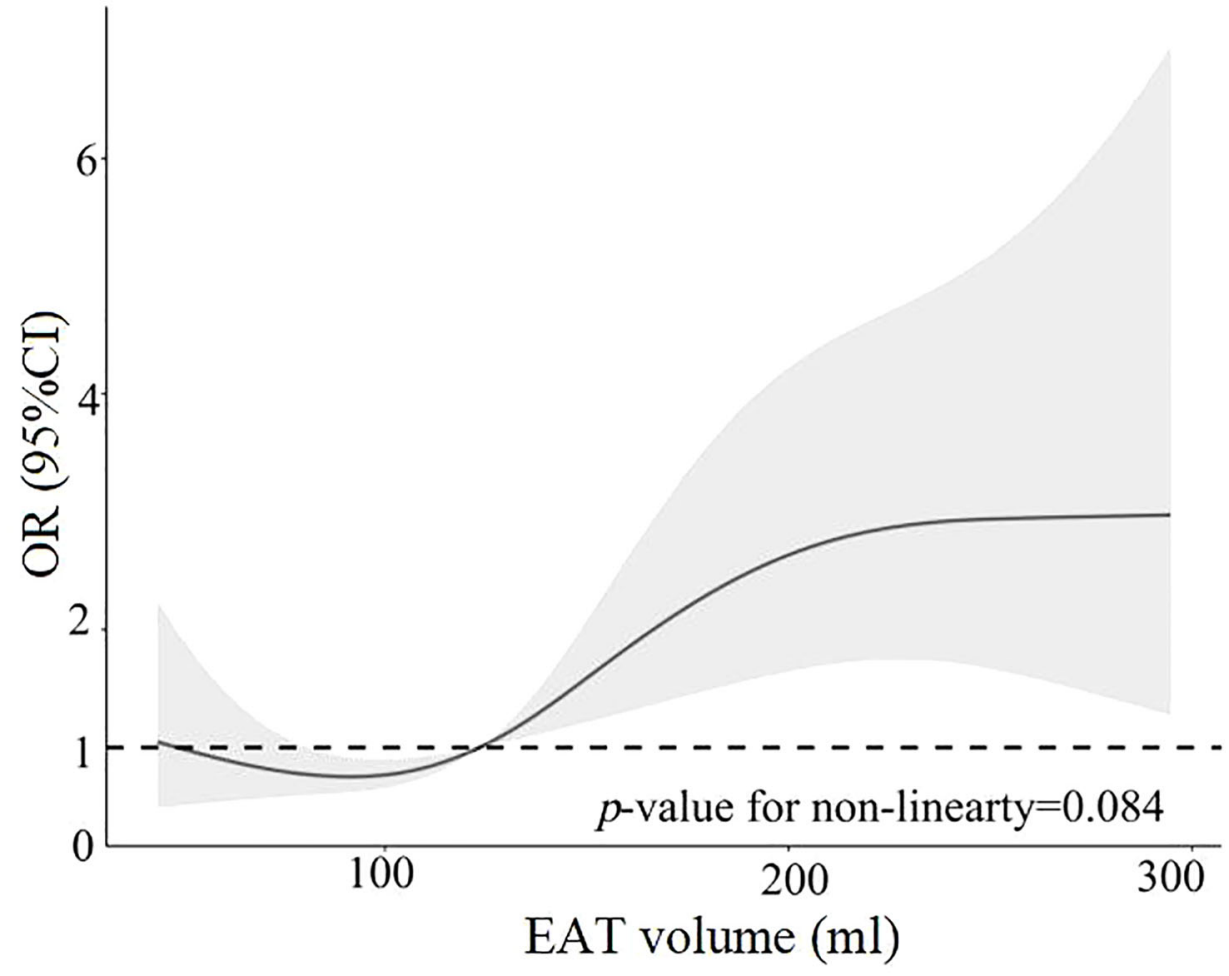
Restricted cubic spline analysis of high PVC burden risk as a function of EAT volume. EAT, epicardial adipose tissue; PVCs, premature ventricular complexes.

**Figure 5:**
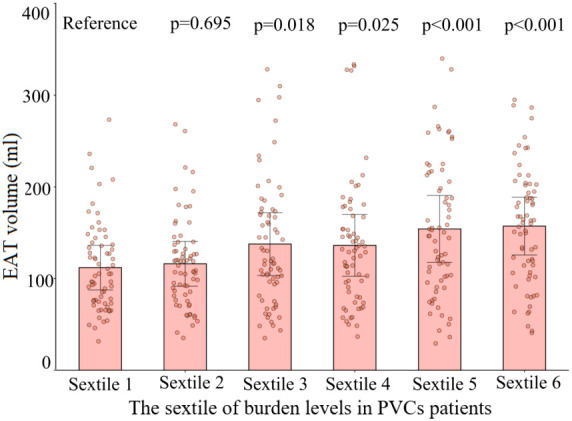
Comparison of the volume of EAT with different burden levels of PVCs. The dots represent measured EAT volume. EAT volumes in sextiles 2 to 6 of PVC burden were compared with those in the sextile 1 group. Sextile 1: burden levels < 12.9%; sextile 2: 12.9% ≤ burden levels < 16.1%; sextile 3: 16.1% ≤ burden levels < 19.3%; sextile 4: 19.3% ≤ burden levels < 24.8%; sextile 5: 24.8% ≤ burden levels < 32.0%; sextile 6: burden levels ≥ 32.0%. EAT, epicardial adipose tissue; PVCs, premature ventricular complexes.

The 402 PVC patients were divided into two groups—those with high burden levels of PVCs and those with low burden levels of PVCs—according to the median burden level of 19.32%. Compared with patients with low burden levels of PVCs, patients with high burden levels had significantly larger age, LAD, and EAT volume and higher proportions of being a smoker, having diabetes mellitus, and having an E/A ratio <1 (**Supplementary Table 2**). Variables included in the univariable analysis showed *p*-value <0.10, and clinical risk factors (fasting blood glucose, total cholesterol) were included in the multivariable analysis, which included age, female gender, smoker, fasting blood glucose, total cholesterol, diabetes mellitus, LAD, E/A ratio <1, and EAT volume (per 10 ml increase). EAT volume (per 10 ml increase; HR: 1.06; 95% CI: 1.01–1.10; *p* = 0.008) remained independently associated with high burden levels of PVCs, as described in **Table 3**. The risk of high PVC burden levels increased with the rising of EAT volume (*p* for non-linearity = 0.084) by RCS, using the median of EAT volume as the reference (123.38 ml), as illustrated in **Figure 4**. Moreover, EAT volume with high PVC burden levels significantly increased in PVC patients with burden levels of sextiles 3–6, with sextile 1 serving as the reference (**Figure 5**).

**Table 3:** Multivariable logistic analysis of risk factors for the burden levels of PVCs.

Variables	OR (95% CI)	*p-*value
Age	1.00 (0.98~1.02)	0.936
Female	1.07 (0.66–1.73)	0.781
Smoker	1.69 (0.90–3.16)	0.101
FPG	0.98 (0.82–1.17)	0.825
TC	0.87 (0.65–1.16)	0.336
DM	1.74 (0.75–4.01)	0.197
LAD	1.03 (0.99–1.07)	0.189
E/A ratio <1	1.08 (0.65–1.80)	0.762
EAT volume (per 10 ml increase)	1.06 (1.01–1.10)	0.008

Variables subjected to univariable analysis showed p-value <0.10, and clinical risk factors (FPG, TC) were included in the multivariable analysis, including age, female gender, smoker, FPG, TC, DM, LAD, E/A ratio <1, and EAT volume (per 10 ml increase).

DM, diabetes mellitus; EAT, epicardial adipose tissue; FPG, fasting blood glucose; LAD, left atrial diameter; PVCs, premature ventricular complexes; TC, total cholesterol; WBC, white blood cell.

## Discussion

There are several major findings in our study. First, EAT volume was increased in frequent PVC patients compared with control patients. The association was independent of other risk factors. Second, the larger EAT volume was independently associated with high PVC burden levels. There was a linear relationship between increased EAT volume and the risk of high PVC burden levels. These findings indicated that EAT volume might be a useful marker to assess the risk of PVCs and high PVC burden levels.

There is no border zone between the EAT and the myocardium, which indicated that EAT plays a vital role in arrhythmias. In the myocardium, a combined effect of structural, electrical, and autonomic remodeling may contribute to arrhythmias (12). The myocardium is affected by some metabolites, which are released from the EAT (13). Non-contrast CT can measure EAT volume to stratify cardiovascular risk with accuracy and reproducibility similar to CCTA (8). EAT volume by non-contrast CT can reduce extra costs or radiation exposure. There are no puncture injuries and contrast agent injection-related complications (14). EAT volume quantification in non-contrast CT is feasible and reliable (15). Our study used sophisticated software to quantify the EAT volume.

An increasing number of studies tried to investigate the relationship between EAT and arrhythmias (13). A meta-analysis study described that large EAT volume increases the prevalence and progression of AF (16). The accumulation of EAT could promote the progression from paroxysmal to persistent AF by prolonging action potential durations (12). Sepehri et al. revealed that a large anterior interventricular groove of EAT thickness is an independent risk factor for the recurrence of ventricular tachycardia in patients following ablation (17). Another study revealed that EAT thickness is larger in patients with frequent PVC ablation failure (18). QRS widening indicates slow ventricular conduction or ventricular hypertrophy, and increased EAT is associated with widened and fragmented QRS waves. The QTc interval represents the time between ventricular depolarization and repolarization. EAT accumulation was found to be associated with prolonged QTc interval. Further studies need to investigate the relationship between EAT and ventricular repolarization.

We considered that there is insufficient direct evidence to assess the relationship between EAT volume and PVCs. Decisions based on baseline PVC burden, rather than on the so-called etiology, seem to be more appropriate for determining treatment modalities (19). Our study first revealed that patients with frequent PVCs had a large EAT volume by non-contrast CT measurement, compared with control patients. Considering that the thickness of EAT cannot accurately reflect the overall level of EAT, CT was used in our study to measure the volume of EAT. Our study suggested that EAT volume, as a non-invasive imaging biomarker, may potentially assess this risk of PVCs.

In our study, male patients with diabetes mellitus, BMI ≥24 kg/m^2^, and E/A ratio <1 had a larger EAT volume in the frequent PVC cohort. Recent studies demonstrated these results (20, 21). Both obesity and type 2 diabetes are important risk factors for AF, possibly because they both cause an expansion of EAT, which is the source of pro-inflammatory adipocytokines that can lead to microvascular dysfunction and fibrosis of the myocardium. In obese and T2D patients, the EAT is thickened and dysfunctional, promoting cardiovascular damage (22). Importantly, the accumulation of cardiac adipose tissue in patients with obesity and type 2 diabetes affects the atria and the ventricles (23). A large EAT volume may be associated with impaired left cardiac diastolic function in PVC patients (24). Accumulation of EAT can lead to adjacent myocardial microcirculation dysfunction and impaired ventricular dilation (23). It indicated that a larger EAT volume might represent higher levels of cardiometabolic risk factors. This also suggested that these cardiovascular risk factors may be risk components for a larger EAT volume (20). Multiple risk factors for PVCs have been shown to act synergistically to promote the development of arrhythmias (25). The mechanisms underlying the relationship between EAT volume and cardiometabolic risk still require more in-depth study. Furthermore, our study revealed the independent association between the burden levels of PVCs and EAT volume. EAT volume may potentially quantify the burden levels of PVCs. EAT may alter the local electrophysiological properties and provide a means of risk assessment.

Several potential mechanisms can be proposed to explain the relationship between EAT volume and PVCs. Firstly, EAT can release inflammatory cytokines. EAT is associated with a high-level inflammation state (26). Second, the accumulation of EAT may cause abnormalities in Ca^2+^ handling and in excitation–contraction coupling, which lead to the disruption of intercellular electrical conduction in myocytes and slow cardiac electrical conduction (12, 27). Another hypothesis linking EAT to PVCs is related to adipose cells. EAT infiltration in the myocardium formats an anatomical obstacle for cardiac excitation, which can promote the degeneration of adjacent cardiomyocytes (28). EAT accumulation could release excessive free fatty acids and promote myocardial lipotoxicity, which leads to mitochondrial dysfunction, and increase reactive oxygen species production, which increases susceptibility to arrhythmias (29). To elucidate these mechanisms, it is necessary to investigate the relationship between the various bioactive substances expressed in EAT and pathological conditions.

Given the important role of EAT in the development of arrhythmias, it is considered a promising therapeutic target. The systematic review and meta-analysis provide evidence that exercise, diet, and bariatric surgery can reduce cardiac adipose tissue (30). In an echocardiographic study of subjects before and after bariatric surgery, weight loss was associated with a 30% reduction in visceral fat area and a 14% reduction in EAT thickness (31). Cardiometabolic drugs have significant benefits in reducing EAT, such as glucagon-like peptide-1 receptor agonists, sodium-glucose cotransporter 2 inhibitors, metformin, and statins, especially for young patients with high BMI (32). Furthermore, we should note that whether the reduction of EAT can confer benefits for patients’ clinical outcomes needs further investigation.

### Limitations

Our study presented some limitations. First, this single-center study was a cross-sectional, observational study, which may lead to selection bias. It is difficult to determine any causal relationship between PVCs and EAT volume. Second, patients with coronary artery disease and AF were excluded from the study, and we considered the close association of these diseases with EAT, which leads to the inaccuracy of the research results. Third, we selected patients with high burden levels of PVCs with more symptoms and impaired cardiac function, limiting the generalizability of the results. However, it is more meaningful to study patients with frequent PVCs, which need more treatment and follow-up. Moreover, our study did not include any reference standard, such as histopathology, to confirm absolute EAT volume. However, we applied a sophisticated CT software for automatic quantification of EAT volume. Finally, we were not able to assess the potential association between PVCs and the ventricular region of EAT. To address this issue, further research may be needed using CMR to quantify the EAT volume in particular areas and investigate the impacts of different EAT areas on PVC patients. Our study could not include all confounding factors, which may alter the power of the statistical analysis and the study’s conclusion. Further prospective studies involving matched groups are needed to confirm the findings.

## Conclusions

EAT volume was larger in frequent PVC patients compared with control patients. A large EAT volume was independently associated with high burden levels of PVCs. EAT volume may be a new mechanism to explain the pathogenesis of PVCs.

## Data availability statement

The raw data supporting the conclusions of this article will be made available by the authors, without undue reservation.

## Ethics statement

The studies involving humans were approved by the First Affiliated Hospital of Zhengzhou University. The studies were conducted in accordance with the local legislation and institutional requirements. The ethics committee/institutional review board waived the requirement of written informed consent for participation from the participants or the participants’ legal guardians/next of kin because this is an observational study that does not involve drug and device interventions.

## Author contributions

ZW, YS, and YC conceived and designed the study. ZW, SJ, JC, HG, and LR participated in the acquisition of data. ZW, SJ, and JC analyzed the data. ZW drafted the manuscript. LS, YS, and YC revised the manuscript. All authors contributed to the article and approved the submitted version.

## Supplementary material

Supplementary Figure 1 Frequent PVCs patient selection and study design. CT, computed tomography; PSM: propensity score matching; PVCs, premature ventricular complexes.

Supplementary Figure 2 Standardized mean differences of different cohorts in this graph. BMI, body mass index; DM. diabetes mellitus; EAT, epicardial adipose tissue; FPG, fasting blood glucose; LAD, left atrial diameter; LVEF, left ventricular ejection fraction; TC, total cholesterol.

Supplementary Figure 3 Receiver operating characteristic curve illustrating the accuracy of EAT volume for frequent PVCs risk. EAT, epicardial adipose tissue; PVCs, premature ventricular complexes.
