# Supplementary material for: The relationship between frequent premature ventricular complexes and epicardial adipose tissue volume
Source: Front Endocrinol (Lausanne). 2023 Sep 26;14:1219890. doi: 10.3389/fendo.2023.1219890 (PMC10562624; doi:10.3389/fendo.2023.1219890)

**Supplementary Table 1.** Correlation of EAT volume with quantitative variables in frequent PVCs cohort.

| Variables of 402 PVCs patients | r | *p-*valu*e* |  |
| --- | --- | --- | --- |
| Age | 0.396 | <0.001 |  |
| BMI | 0.388 | <0.001 |  |
| WBC | 0.111 | 0.026 |  |
| FPG | 0.143 | 0.004 |  |
| HS-CRP | 0.056 | 0.306 |  |
| TC | 0.052 | 0.297 |  |
| TG | 0.149 | 0.003 |  |
| LVEF | -0.105 | 0.036 |  |
| LAD | 0.322 | <0.001 |  |

Abbreviations: BMI, body mass index; EAT, epicardial adipose tissue; FPG, fasting blood glucose; HS-CRP, high-sensitivity C-reactive protein; LAD, left atrial diameter; LVEF, left ventricular ejection fraction; PVCs, premature ventricular complexes; TC, total cholesterol; TG, triglycerides; WBC, white blood cells.**Supplementary Table 2.** Baseline parameters according to the burden levels of frequent PVCs.

| Variables | High PVCs burden | Low PVCs burden | *p*-value |
| --- | --- | --- | --- |
| All patients (n=402) | n=201 | n=201 |  |
| Age | 49.3 ± 12.7 | 46.5 ± 11.6 | 0.023 |
| Female | 96 (47.8) | 113 (56.2) | 0.090 |
| Smoker | 43 (21.4) | 24 (12.0) | 0.012 |
| Drinker | 33 (16.4) | 26 (12.9) | 0.324 |
| BMI | 24.8 ± 3.2 | 24.9 ± 3.6 | 0.752 |
| Hypertension | 27 (13.4) | 39 (19.4) | 0.106 |
| DM | 22 (10.9) | 11 (5.5) | 0.046 |
| WBC | 6.3 ± 1.8 | 6.3 ± 1.6 | 0.759 |
| FPG | 5.1 ± 1.3 | 5.0 ± 1.3 | 0.331 |
| TC | 4.3 ± 0.7 | 4.3 ± 0.7 | 0.392 |
| TG | 1.2 (0.9-1.8) | 1.2 (0.9-1.8) | 0.857 |
| LAD | 34.4 ± 6.2 | 32.8 ± 5.6 | 0.005 |
| LVEF | 62.2 ± 3.8 | 62.6 ± 3.2 | 0.287 |
| E/A ratio<1 | 111 (55.2) | 88 (43.8) | 0.022 |
| EAT volume | 149.2 ± 68.3 | 121.9 ± 57.0 | <0.001 |

Note: The PVCs burden level was a categorical value according to the median (19.32%). The high PVCs burden levels group was defined as ≥ 19.32%; The low PVCs burden levels group was defined as < 19.32%. BMI, body mass index; DM. diabetes mellitus; EAT, epicardial adipose tissue; FPG, fasting blood glucose; LAD, left atrial diameter; LVEF, left ventricular ejection fraction; PVCs, premature ventricular complexes; TC, total cholesterol; TG, triglycerides; WBC, white blood cell.

**Supplementary Figure 1.** Frequent PVCs patient selection and study design. CT, computed tomography; PSM: propensity score matching; PVCs, premature ventricular complexes.


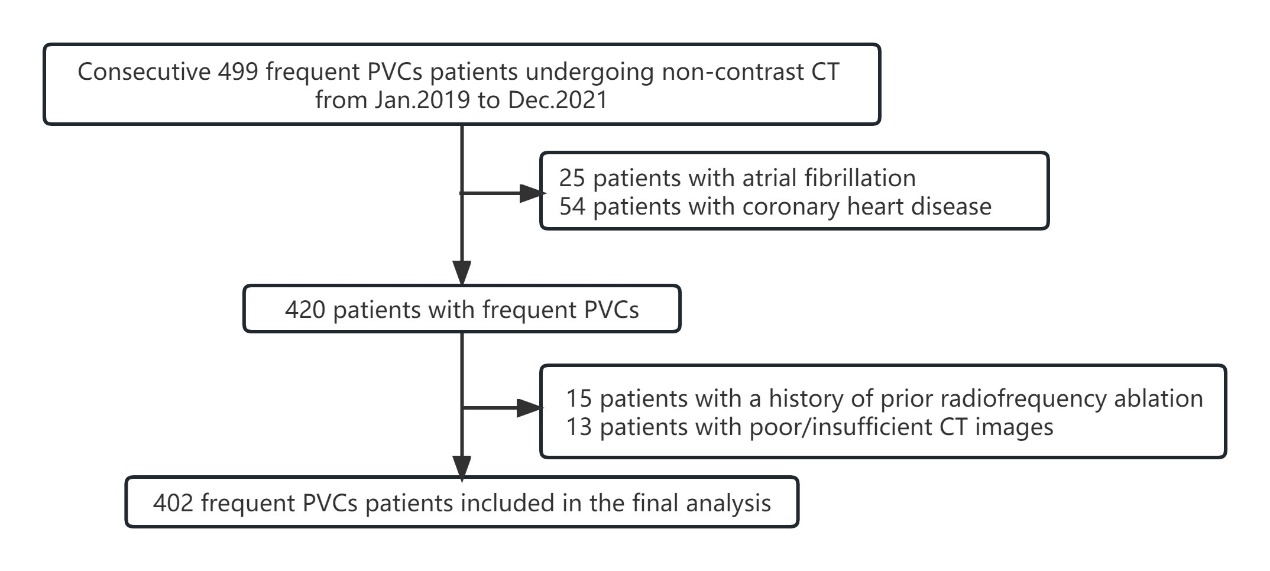


**Supplementary Figure 2.** Standardized mean differences of different cohorts in this graph. BMI, body mass index; DM. diabetes mellitus; EAT, epicardial adipose tissue; FPG, fasting blood glucose; LAD, left atrial diameter; LVEF, left ventricular ejection fraction; TC, total cholesterol.


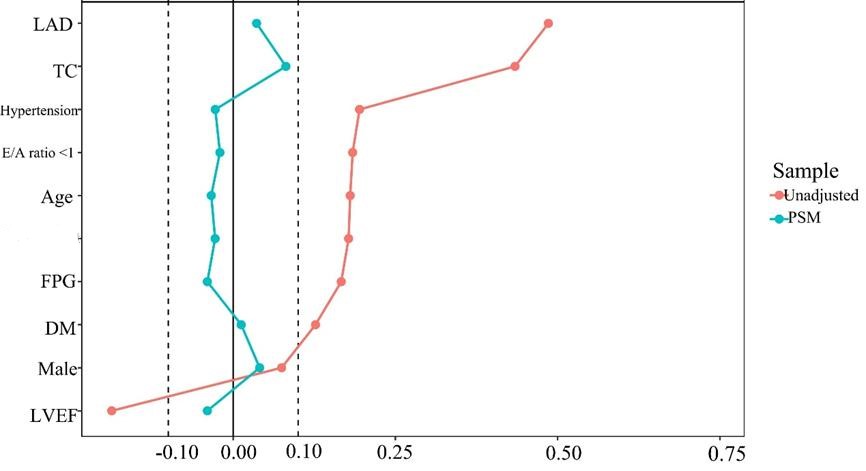

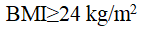


**Supplementary Figure 3.** Receiver operating characteristic curve illustrating the accuracy of EAT volume for frequent PVCs risk. EAT, epicardial adipose tissue; PVCs, premature ventricular complexes.


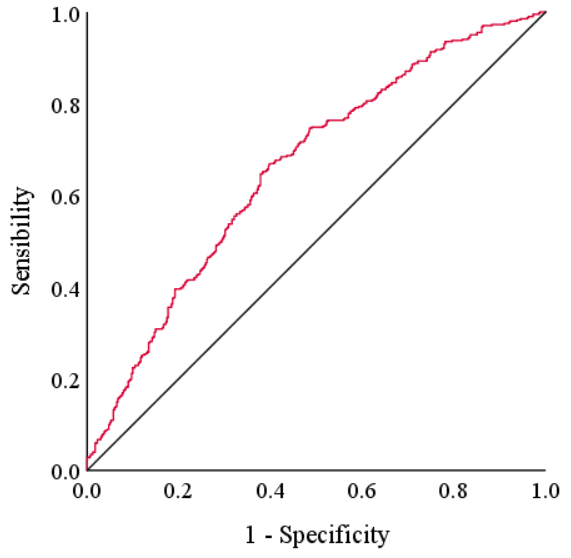

Supplement: Supplementary Figure 1 — Frequent PVCs patient selection and study design. CT, computed tomography; PSM: propensity score matching; PVCs, premature ventricular complexes. [file DataSheet_1.docx]
